# Yiqi Huayu Jiedu Decoction reduces colorectal cancer liver metastasis by promoting N1 neutrophil chemotaxis

**DOI:** 10.3389/fimmu.2025.1530053

**Published:** 2025-02-27

**Authors:** Hua-Jian Zhou, Bai-Xiang Mu, Meng-Chao Wen, Qi Zhao, Yuanxiang Li, Wen-Xuan Zhao, Hong-Ye Yin, Shuai Ren, Jin-Yong Zhou, Min Chen

**Affiliations:** ^1^ Affiliated Hospital of Nanjing University of Chinese Medicine, Jiangsu Province Hospital of Chinese Medicine, Nanjing, Jiangsu, China; ^2^ Central Laboratory, Jiangsu Province Hospital of Chinese Medicine, Affiliated Hospital of Nanjing University of Chinese Medicine, Nanjing, Jiangsu, China; ^3^ Department of Radiology, Affiliated Hospital of Nanjing University of Chinese Medicine, Nanjing, China; ^4^ Jiangsu Province Key Laboratory of Tumor Systems Biology and Chinese Medicine, Jiangsu Province Hospital of Chinese Medicine, Affiliated Hospital of Nanjing University of Chinese Medicine, Nanjing, Jiangsu, China

**Keywords:** colorectal cancer, liver metastasis, Yiqi Huayu Jiedu Decoction, CXCL1, neutrophils

## Abstract

**Objective:**

To observe the inhibitory effect and potential mechanism of Yiqi Huayu Jiedu Decoction (YHJD) on liver metastasis of colorectal cancer (CRC).

**Methods:**

We compared the changes of liver weight and liver index before and after YHJD treatment in CRC liver metastasis mouse models. HE staining was employed to observe the pathological changes in mouse liver tissue sections. Flow cytometry was used to analyze the number and marker of neutrophils treated with YHJD. Transcriptomics, proteomics, and multiplex cytokine array analyses were conducted to further verify the role of YHJD on CXCL1. Differential gene analysis was performed to further explore the mechanism by which YHJD inhibits liver metastasis of CRC.

**Results:**

Animal studies demonstrated that YHJD reduces liver metastases. Flow cytometry results revealed that YHJD promotes N1 neutrophils in liver. Combining multi-omics and multiple cytokine arrays, we observed a significant increase in the expression of CXCL1 in the liver and plasma. GO and KEGG enrichment analyses indicated that YHJD may regulate the chemotaxis of neutrophils to inhibit the liver metastasis of CRC by participating in the regulation of cell adhesion molecule binding, adhesion protein binding, and multiple metabolic pathways.

**Conclusions:**

YHJD inhibits CRC liver metastasis by upregulating CXCL1, thereby promoting N1 neutrophil chemotaxis towards the liver, and concurrently raising the expression of N1 neutrophil markers.

## 1 Introduction

Colorectal cancer (CRC) ranks as the third most common cancer worldwide and has the second-highest mortality rate among all cancers (1). Approximately 20% of CRC patients develop metastasis, with the liver being the most frequent site of metastasis (2). Liver metastasis is the primary cause of mortality among patients with CRC (3). Despite significant advancements in chemotherapy and surgical interventions, CRC patients who develop metastases have a worse prognosis (4). Given the limitations of current treatments, there is an imperative need for novel therapeutic strategies for CRC liver metastasis. Our study provides an insight for the research and treatment of liver metastasis of intestinal cancer in terms of improving the tumor microenvironment (TME) by traditional Chinese medicine decoctions.

Neutrophils, as innate immune cells, constitute the host’s initial line of defense against pathogens (5). The dual role of neutrophils in both tumor prevention and facilitation is partially recognized (6). N1 neutrophils function as pro-inflammatory agents and tumor growth suppressors (7). They exert cytotoxic effects either directly or through antibody-dependent mechanisms (8). In addition, N1 neutrophils can interact with a variety of immune cells to inhibit tumor growth and metastasis (9–13). N1 neutrophils can amass cytotoxic mediators within the organ of origin, thereby restricting tumor metastasis (14). Furthermore, N1 neutrophils increase NADPH oxidase activity in neutrophils, killing tumor cells by generating reactive oxygen species (15). N1 neutrophils are closely related to pro-inflammatory or immune-stimulating cytokine release activity (16). Due to their role in suppressing tumor cells, N1 neutrophils have been used in cellular immunotherapy for various tumors (17–20).

Chemokines are produced by leukocytes in concert with histiocytes. By binding to receptors, they have a demonstrated effect on tumor metastasis and progression (21). Chemokines are involved in the migration of immune cells, which is a necessary step for the activation of effective anti-tumor immunity (22). In response to chemokines and cytokines, neutrophils migrate towards the TME. Neutrophils express many chemokine receptors, such as CXCR1 and CXCR2 (23). C-X-C motif chemokines are known to mediate neutrophil aggregation, with CXC ELR^+^ chemokines, such as CXCL1, acting preferentially on neutrophils (24). Neutrophils infiltrate into tumor tissue following the C-X-C chemotaxis axis to adapt to the requirements of the TME (25). Therefore, neutrophil chemotactic function could impact CRC liver metastasis.

Yiqi Huayu Jiedu Decoction (YHJD), derived from the renowned Chinese traditional formula Si-Jun-Zi Decoction, is composed of various medicinal herbs, including Radix Astragali, Codonopsis pilosula, Rhizoma Atractylodis Macrocephalae, Dioscoreae Rhizome, Poria, Glycyrrhizae Radix et Rhizoma, Mume Fructus, Sparganii Rhizoma, Curcumae Rhizoma, Agrimoniae Herba, and patrinia. Previous experiments conducted by our team have demonstrated that the full dose modified Si-Jun-Zi Decoction significantly increases the number of macrophages in the spleen and attenuates CRC liver metastasis in the nude mice model (26). Additionally, YHJD exerts inhibitory effects on CRC liver metastasis by enhancing NK cell functions (27). Moreover, it has been observed to inhibit postoperative metastasis and recurrence in patients with gastric cancer (28). Based on these findings, we explored the role of YHJD in regulating neutrophil chemotaxis by acting on CXCL1 and thereby modulating neutrophil chemotaxis to further elucidate the multifaceted mechanisms by which YHJD inhibits CRC liver metastasis.

## 2 Materials and methods

### 2.1 Animals

Male BALB/c mice (18-20g, SPF grade) were procured from Sibeifu Biology Technology Co., Ltd., with a production license number: SCXK (Su) 2022-0006. The experimental mice were housed in the barrier environment animal room at the Animal Center of Jiangsu Provincial Hospital of Chinese Medicine, maintained at a room temperature of 22-24°C and humidity of 40%-60%. The animal facility is licensed under License number: SYXK (Su) 2022-0070. All animal experiments were conducted following approval from the Ethics Committee of the Affiliated Hospital of Nanjing University of Chinese Medicine (Ethics No. 2023DW-021-01).

### 2.2 Drug preparation

YHJD Decoction Ingredients: Radix Astragali (Batch number: 2304435301), Glycyrrhizae Radix et Rhizoma (Batch number: 2308142302), Codonopsis pilosula (Batch number: 2305023302), Rhizoma Atractylodis Macrocephalae (Batch number: 2201242391), Poria(Batch number: 2310092301), Dioscoreae Rhizome (Batch number: 2310011301), Mume Fructus (Batch number: 2302485301), Sparganii Rhizoma (Batch number: 2309306301), Curcumae Rhizoma (Batch number: 2306428301), Agrimoniae Herba (Batch number: 2305103301), Patrinia (Batch number: 2303185301). These ingredients were provided by Tian Jiang Pharmaceutical Co., Ltd. The entire formula granules were dissolved in 200 mL of double-distilled water, stirred thoroughly, heated to boiling, and concentrated in the herbal solution to a concentration of 2 g herb/mL.

### 2.3 Main reagents and instruments

RPMI 1640 medium basic (Thermo Fisher, Cat: C11875500BT, China); Fetal bovine serum (Biological Industries, Cat: C04001, Israel); Phosphate Buffered Saline (Servicebio, Cat: G4207, China); Antibodies: Brilliant Violet 421™ anti-mouse/human CD11b (BioLegend, Cat: 101251, Lot: B303400, USA), APC anti-mouse Ly-6G (BioLegend, Cat: 127613, Lot: B296100, USA), PE-ICAM-1 (eBioscience, Cat: 12-0542-82, Lot: 2186512, USA), Anti-Mo CD45 (Invitrogen, REF: 11-0451-82, Lot: 2607666, USA); Mouse Cytokine Array Q5 kit (Raybiotech, Inc, No: QAM-CYT-5-1, China); ChemiDoc MP gel imaging system (BIO-RAD, USA); BD FACSCelesta flow cytometer (BD, San Jose, CA, USA); Illumina NovaSeq 6000 (Illumina, CA, USA); Bioanalyzer 2100 (Agilent, CA, USA).

### 2.4 Cell culture

The mouse CRC cell line CT26 was purchased from the Shanghai Institute of Cell Research, Chinese Academy of Sciences. The cells were cultured in RPMI 1640 medium supplemented with 10% fetal bovine serum, streptomycin, and penicillin. The cell culture incubator was maintained at 37°C with 5% carbon dioxide.

### 2.5 CRC liver metastasis model

The mice were randomly divided into three groups: the control group, the model group, and the YHJD group. Based on our previous experimental experience, mice were given a gavage dose of 0.2 mL/20g body weight twice a day. Equivalent amounts of saline were given to the Control and Model groups. For modeling, mice were intraperitoneally anaesthetized with a 1% sodium pentobarbital solution under sterile conditions. A single-cell suspension containing 2×10^5^ mouse colon cancer CT26 was taken and inoculated into the spleen of mice in a volume of 20 μL. The mice were randomly grouped the following day and received 3 weeks of treatment. Two hours after the last administration, the mice were sacrificed. Their livers were immediately photographed and weighed. Livers were cut into small pieces and preserved for subsequent experimental manipulations.

### 2.6 Hematoxylin-eosin staining

Liver specimens from mice in each group were fixed in 10% formaldehyde for 24 hours. Pathological sections were prepared using the HE staining method, and the formation of liver metastases was observed by scanning the sections.

### 2.7 Flow cytometry analysis

A portion of liver tissue was dissected and gently minced, then filtered through a 40 μm cell strainer to obtain a single-cell suspension. Flow cytometry antibody staining was conducted, wherein all leukocytes were initially selected by CD45^+^ to exclude liver cells. Neutrophils were subsequently identified using specific antibodies. Flow analysis was performed using a BD FACSCelesta flow cytometer, and the data were analyzed using FlowJo (version: 10.8.1). The following parameters were assessed: (1) Neutrophil percentage, determined by the percentage of CD11b^+^, Ly6G^+^ double-positive cells; (2) The MFI of N1 neutrophils marker (ICAM-1^+^) among the aforementioned double-positive cells.

### 2.8 Multiplex cytokine array

We conducted various cytokine analyses on the plasma of mice using the mouse cytokine array Q5 assay kit. Subsequently, protein expression levels in the plasma were detected according to the instructions, and proteins that underwent significant changes were identified.

### 2.9 Transcriptomics sequencing

After isolating and purifying RNA from the liver samples, we conducted quality control to test the RNA’s integrity. The mRNA containing PolyA (polyadenylate) was specifically captured through two rounds of purification. The captured mRNA was then fragmented. The fragmented RNA was synthesized into cDNA by reverse transcriptase, followed by two-strand synthesis. The complex double strand of DNA and RNA was converted into a double-stranded DNA, with dUTP Solution added to make the ends of the double-stranded DNA flat. The fragment size was then screened and purified using magnetic beads. The second strand was digested with UDG enzyme, and then PCR was used to form a library with a fragment size of 300 bp ± 50 bp. Finally, bipartite sequencing was performed.

### 2.10 Proteomics experiment

We commissioned ShangHai Bioprofile to perform proteomics analyses. In brief, after the cells lysed, we homogenized them. The samples were then centrifuged at 12,000g for 15 minutes to remove any undissolved cellular debris. The supernatant was collected and quantified using the BCA Protein Assay Kit (Bio-Rad, USA). Next, the samples were ultrafiltered repeatedly using 200 µL of UA buffer and then centrifuged. Iodoacetamide was added to the UA buffer to block reduced cysteine residues, and the samples were incubated in the dark for 20 minutes. The protein suspension was digested with 4 µg of trypsin in 40 µL of 25 mM NH_4_HCO_3_ solution overnight at 37°C, and the peptides were collected in the filtrate. The peptide concentration was determined. The peptides were then labeled with TMT reagent, and the TMT-labeled peptide mixture was fractionated. Fractions were dried and used for nano LC-MS/MS analysis. Subsequently, LC-MS analysis was carried out, and the resulting LC-MS/MS raw files were imported into Proteome Discoverer 2.4 software (version 1.6.0.16) for data interpretation and protein identification against the database. Ion intensities were reported using TMT for quantification.

### 2.11 Bioinformatics analysis

The differentially expressed genes obtained from transcriptomics and proteomics analyses were subjected to Venny2.1 to identify common differential genes. These common differential genes were then inputted into Metascape (29) for Gene Ontology (GO) and Kyoto Encyclopedia of Genes and Genomes (KEGG) enrichment analysis. Additionally, CXCL1 and Fas obtained from the preliminary data analysis were entered into the Kaplan-Meier Plotter (30) (https://kmplot.com/analysis/, accessed on 22 April 2024) online platform for survival analysis and treatment sensitivity analysis, as well as into the TIMER (31) (https://timer.cistrome.org/, accessed on 22 April 2024) online platform for immune-related neutrophil immune infiltration analysis. Proteomics analysis was performed to identify genes that were significantly up-regulated and down-regulated (fold change >1.25 or <0.8) following intervention with the herbal complex. The significantly up-regulated and down-regulated genes were then entered into the String database for protein interaction analysis.

### 2.12 Statistical analysis

The experimental data were statistically processed using GraphPad Prism (version 9.5.0). The data was analyzed using t-test or one-way ANOVA test for further analysis. Differences were considered statistically significant at P < 0.05.

## 3 Results

### 3.1 YHJD inhibits liver metastasis of CRC in mice

**Figure 1:**
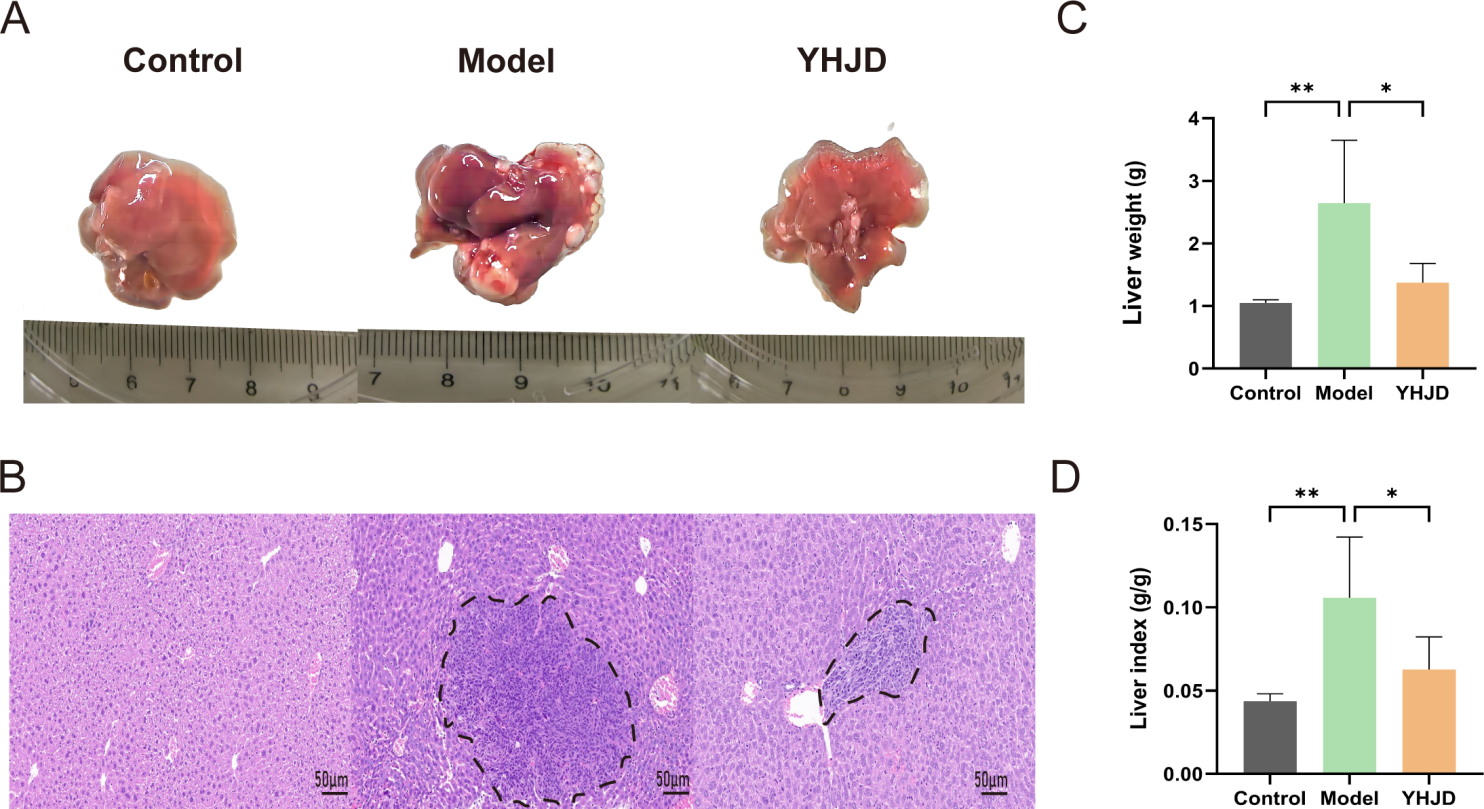
YHJD significantly reduced liver metastasis of CRC in mice (n: Control=6, Model=3, YHJD=3). **(A)** Representative images of mouse livers in each group. **(B)** Representative HE-stained images of mouse livers in the three groups. Tumor tissue is indicated by the black dashed line. Scale bar = 50 μm (×20). **(C)** Statistical plot of liver weight in the three groups of mice. **(D)** Graph of liver index statistics in the three groups of mice. Values are expressed as mean ± SD. *P<0.05, **P<0.01 vs. Model.

To evaluate the impact of YHJD on CRC liver metastasis, we established a liver metastasis model by implanting tumor cells into the spleen. Liver images and HE staining of mice revealed that, compared to the Model group (**Figures 1A, B**), the extent of liver metastasis in mice treated with YHJD was significantly reduced. Statistical analysis demonstrated that following YHJD treatment (**Figures 1C, D**), both liver weight and liver index (=Liver weight/body weight) exhibited a significant decrease.

### 3.2 Effects of YHJD on neutrophils

**Figure 2:**
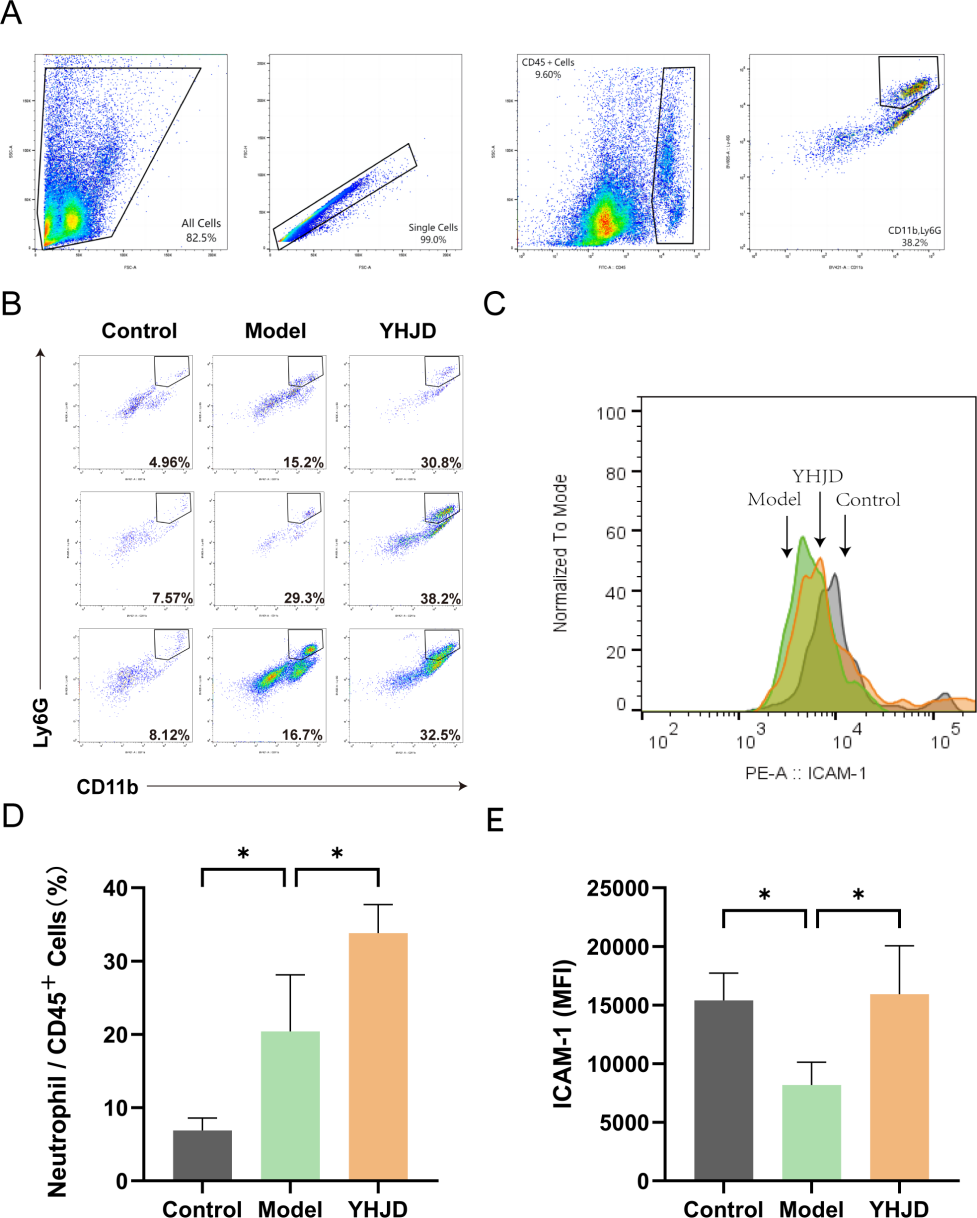
YHJD promotes neutrophil chemotaxis into the liver and increases the percentage of N1 neutrophils (n = 3 per group). **(A)** Flow chart of flow cytometry analysis. **(B)** Representative graphs of the percentage of neutrophils. **(C)** Representative plot of peak fluorescence of ICAM-1. **(D)** Graph showing neutrophils as a percentage of CD45^+^ cells. **(E)** MFI statistics graph for ICAM-1. Values are expressed as mean ± SD. *P<0.05 vs. Model.

We employed flow cytometry to investigate the impact of YHJD on neutrophils in the liver, particularly focusing on N1 neutrophils. The flow cytometry analysis of neutrophils and the corresponding results are depicted in (**Figures 2A, B**). The findings indicated that the percentage of liver neutrophils in mice treated with YHJD was 33.83% ± 5.48%, which was significantly higher than that in the Model group (20.40% ± 7.74%) (**Figure 2C**). Additionally, the mean fluorescence intensity (MFI) of N1 neutrophils was 15402 ± 2328 in the control group, 8178 ± 1942 in the Model group, and 15932 ± 4133 in the YHJD group (**Figures 2D, E**). These results demonstrate that compared to the Model group, YHJD significantly increased the proportion of N1 neutrophils in the livers of mice with CRC liver metastasis.

### 3.3 Effect of YHJD on CXCL1 and Fas in the liver

**Figure 3:**
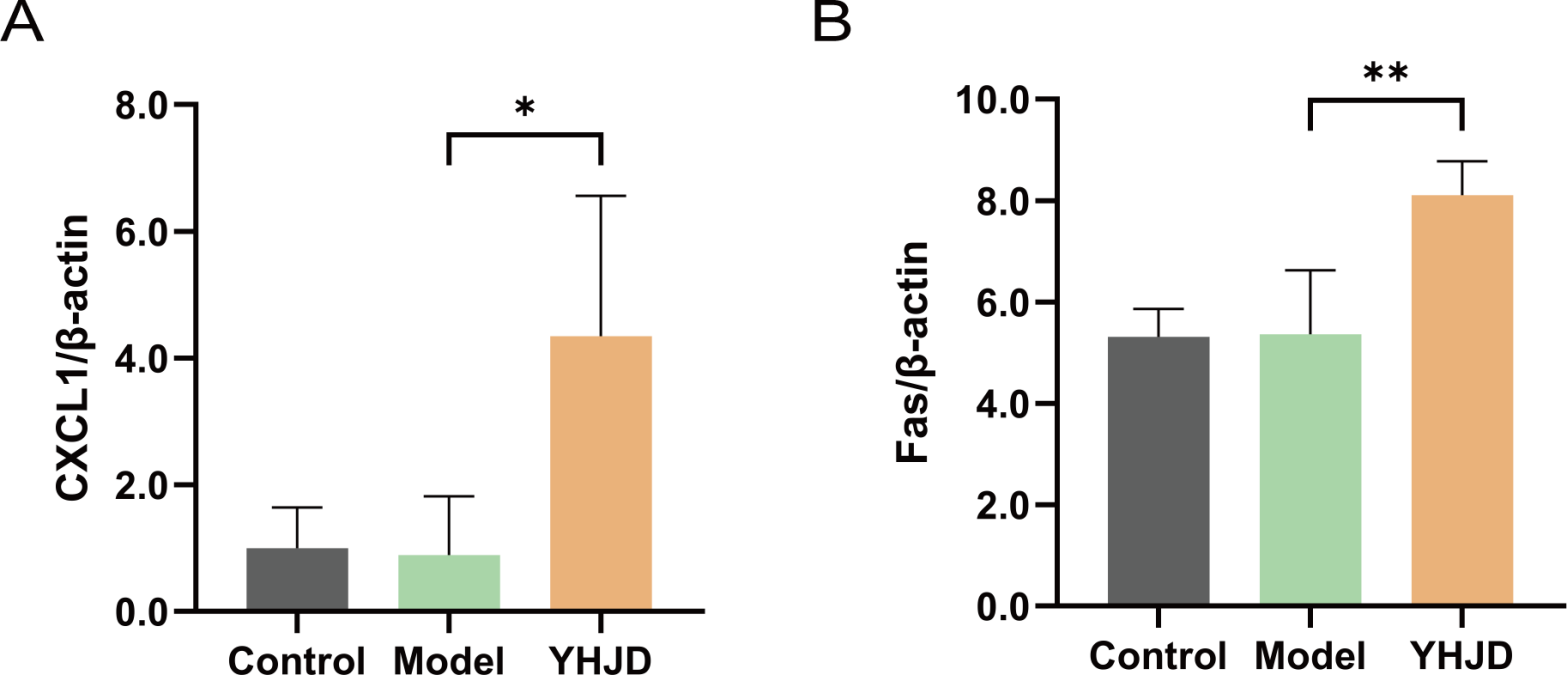
YHJD increases RNA levels of CXCL1 and Fas in the liver from sequencing results (n: Control=4, Model=3, YHJD=4). **(A)** Quantitative results of CXCL1 in liver tissue samples. **(B)** Quantitative results of Fas in liver tissue samples. Values are expressed as mean ± SD. *P<0.05, **P<0.01 vs. Model.

To elucidate the mechanism of action of YHJD on neutrophils, we analyzed mouse liver mRNA. Our results revealed that the level of hepatic CXCL1 was increased in the group of mice treated with the Chinese medicine compared to the model group (**Figure 3A**). Furthermore, we analyzed Fas, a representative marker of N1 neutrophils, and found its expression to be increased in the liver (**Figure 3B**). YHJD was shown to elevate the levels of both CXCL1 and Fas in the liver.

### 3.4 Effect of YHJD on CXCL1 in plasma

**Figure 4:**
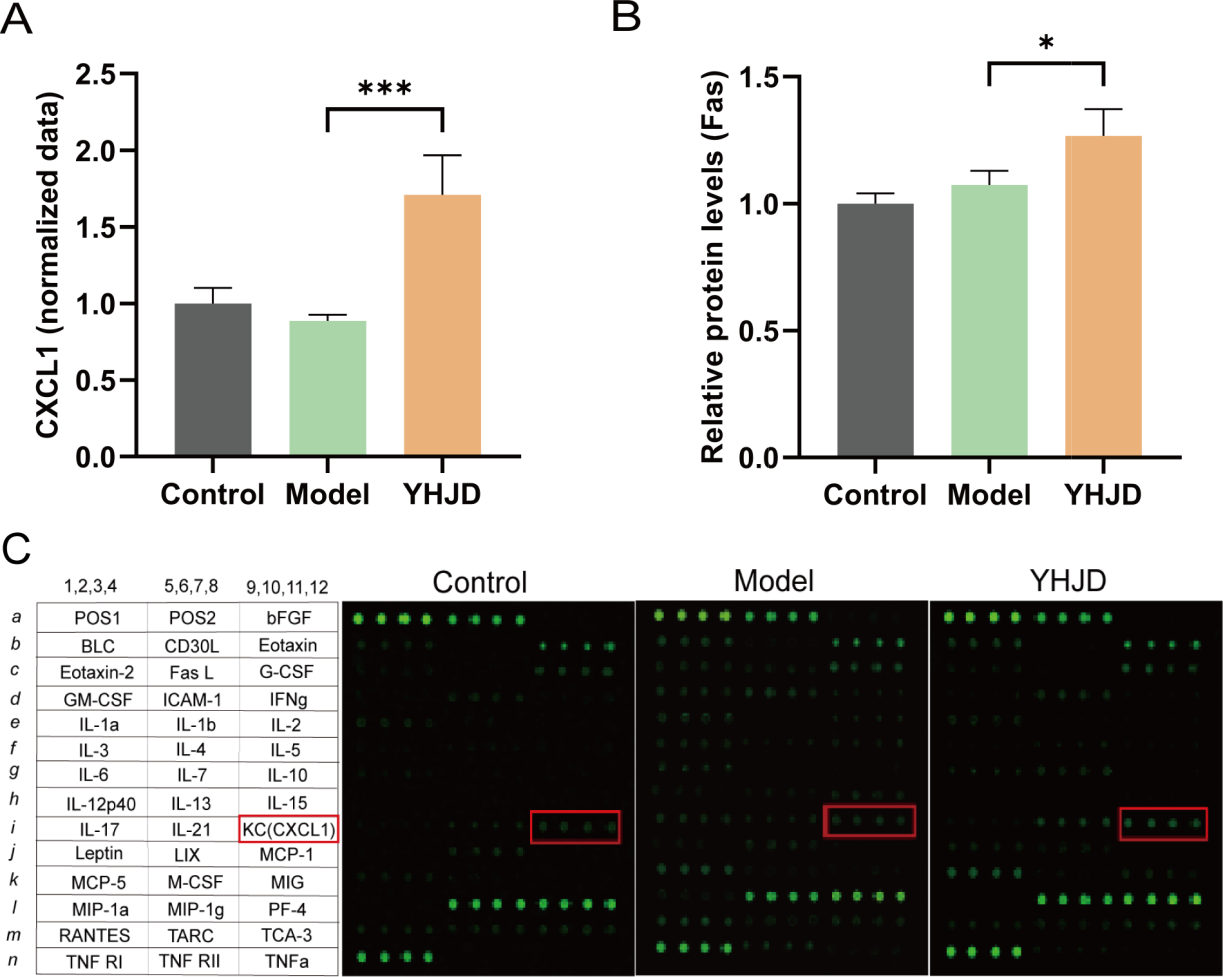
YHJD increases the protein levels of CXCL1 and Fas. **(A)** Statistical graph of CXCL1 plasma cytokines protein expression(n: Control=4, Model=3, YHJD=4). **(B)** Statistical graph of Fas relative protein levels (n: Control=4, Model=3, YHJD=3). **(C)** Name and layout of the 42 proteins and image of plasma cytokines in the three groups of mice. Values are expressed as mean ± SD. *P<0.05, ***P<0.001 vs. Model.

We performed protein-level assays on samples from mice. The results indicated a notable increase in CXCL1 levels in the plasma following YHJD treatment (**Figure 4A**). Relative protein levels of Fas were also significantly increased after YHJD treatment compared to Model (**Figure 4B**). We analyzed a total of 42 proteins and performed layout. Analysis of cytokines in mouse plasma showed a significant increase in the fluorescence intensity of CXCL1 after YHJD treatment (**Figure 4C**).

### 3.5 Differential protein analysis for proteomics

**Figure 5:**
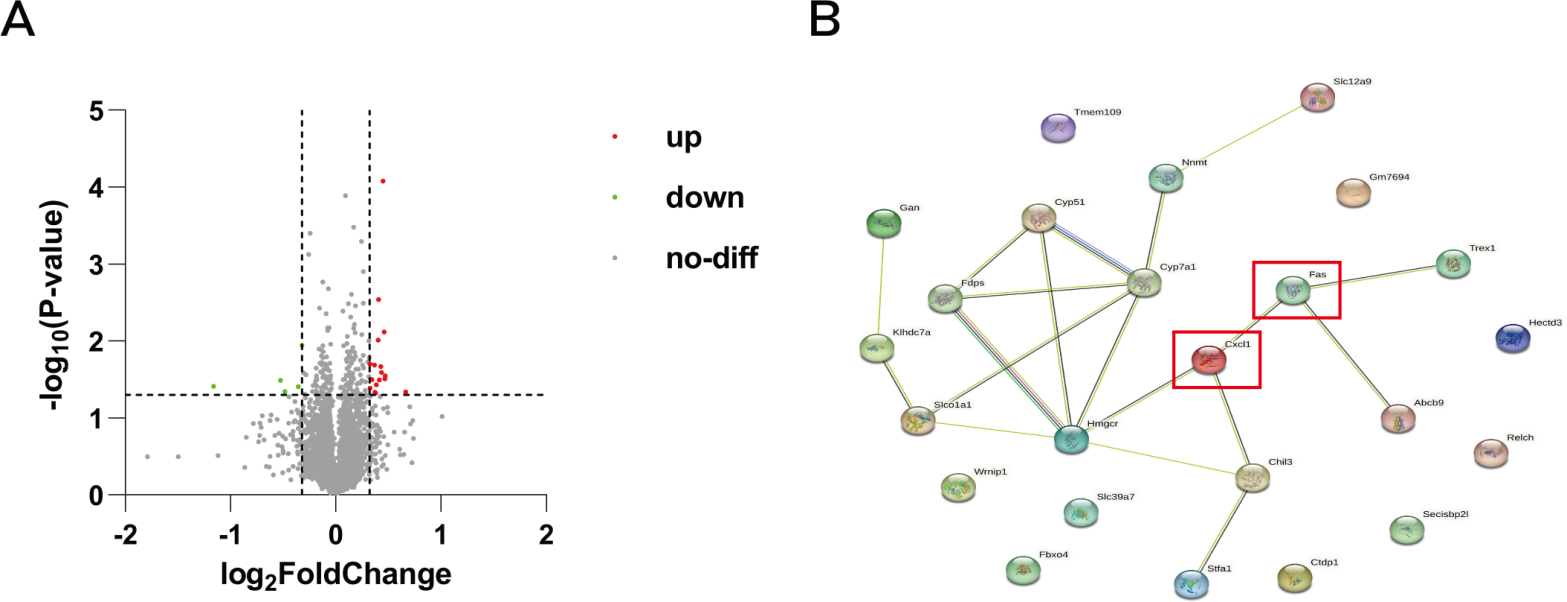
Volcano map and protein interactions network map. **(A)** Volcano plot of differentially expressed genes in proteomic analysis: red dots represent upregulated genes, green dots represent downregulated genes, and gray dots represent genes with no differential changes. **(B)** Protein interaction network of differentially expressed genes with CXCL1 and Fas.

From the proteomics results, we initially identified 5020 proteins. Among these, we pinpointed 188 proteins that exhibited statistically significant differences, with Fold Change >1.25 or Fold Change <0.8 used as the criteria. Ultimately, we identified a total of 22 differential proteins that were significantly up-regulated after herbal treatment (**Figure 5A**). Subsequently, we analyzed these 22 differential proteins for protein interactions with Fas and CXCL1 (**Figure 5B**). Chitinase-like protein 3 (Chil3) was found to interact with CXCL1. This interaction suggests a potential link between Chil3 and CXCL1 in the context of CRC.

### 3.6 Transcriptomics and proteomics analysis

**Figure 6:**
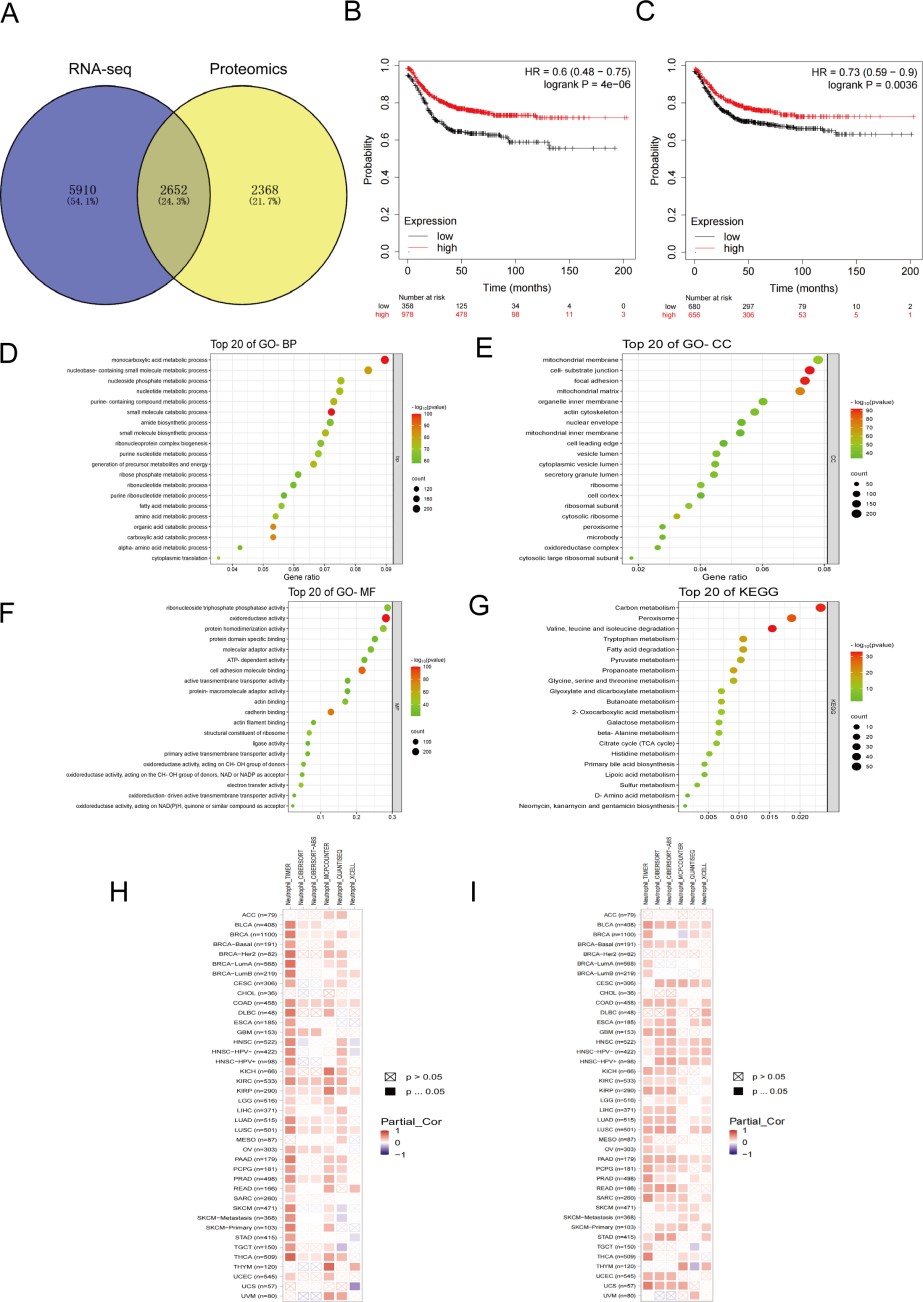
Bioinformatics analysis related to the inhibition of liver metastasis by YHJD. **(A)** Venn diagrams of transcriptomics and proteomics differential genes. **(B)** Survival curves with differential expression of CXCL1 in CRC patients. **(C)** Survival curves with differential expression of Fas in CRC patients. **(D-G)** Bubble plot of GO and KEGG enrichment analysis. **(H)** Heat map of correlation between CXCL1 expression and neutrophil infiltration. **(I)** Heat map of correlation between Fas expression and neutrophil infiltration. The horizontal axis represents different malignant tumors, and the vertical axis represents data from different databases.

The intersection of transcriptomics and proteomics differential genes revealed a total of 2652 differentially expressed genes (**Figure 6A**). Prognostic analysis conducted on CRC patients with varying expression levels of CXCL1 (**Figure 6B**) and Fas (**Figure 6C**) using the Kaplan-Meier Plotter database indicated that high expression levels of CXCL1 and Fas were associated with higher survival rates in CRC patients. GO and KEGG enrichment analyses of these differentially expressed genes revealed their involvement in various Biological Processes (**Figure 6D**), such as small molecule catabolic process, monocarboxylic acid metabolic process, organic acid catabolic process, carboxylic acid catabolic process, and nucleobase-containing small molecule metabolic process. Regarding Cellular Components (**Figure 6E**), the differential genes were primarily associated with cell-substrate junction, focal adhesion, mitochondrial matrix, cytosolic ribosome, and mitochondrial membrane. Molecular Functions (**Figure 6F**) enriched by these genes included oxidoreductase activity, cell adhesion molecule binding, cadherin binding, electron transfer activity, and structural constituent of ribosome. Moreover, the enrichment analysis revealed the involvement of the differential genes in regulating pathways such as Valine, leucine, and isoleucine degradation, sulfur metabolism, propanoate metabolism, butanoate metabolism, D-amino acid metabolism, and others (**Figure 6G**). The analysis of immune infiltration in the TME for CXCL1 (**Figure 6H**) and Fas (**Figure 6I**) revealed an intriguing finding: there was a positive correlation between the expression levels of CXCL1 and Fas and the infiltration of neutrophils. In other words, higher expression levels of CXCL1 and Fas were associated with more pronounced neutrophil infiltration in the TME.

## 4 Discussion

Liver metastasis is the primary cause of death in CRC patients, demonstrating high heterogeneity and an imbalance in anti-tumor immunity within the TME (32). Our study aimed to elucidate the chemotactic effect of YHJD on N1 neutrophils for its anti-tumor effect. Animal experiments have demonstrated that YHJD effectively reduces the number of liver metastases in CRC and exhibits a certain therapeutic effect on CRC. CXCL1 has been identified as a key player in neutrophil recruitment (33). CXCL1, known for its chemotactic effect on neutrophils, is promoted by YHJD in liver tissues to induce the chemotaxis of anti-tumor neutrophil infiltration, potentially reducing liver metastasis in CRC patients. Both CRC mouse liver mRNA and plasma analysis indicate that CXCL1 is a primary target of YHJD. YHJD promotes the formation of an anti-tumor immune microenvironment, as evidenced by the increased entry of N1 neutrophils into the liver and the elevation of representative factors of N1 neutrophils, thereby inhibiting liver metastasis of CRC.

Neutrophils constitute the majority of inflammatory cells in solid tumors (15). They have been demonstrated to act as tumor suppressors at various maturation stages (14, 34). In the early stages of tumorigenesis, neutrophils release cytokines, reactive oxygen species, membrane perforating agents, and soluble factors to promote an inflammatory response and exhibit greater cytotoxicity, producing more TGF-β and NO to eliminate tumor cells (35, 36). Chemokines and cytokines guide neutrophils to migrate and aggregate towards solid tumors, where high-density mature neutrophils have been shown to kill tumor cells (37). Neutrophils exhibit strong plasticity and different phenotypes in different tumors (38). Notably, the receptor for advanced glycation end receptor mediates the recognition of tumor cells by neutrophils, enabling effective tumor cell killing (39). Neutrophils also promote the effect of CD8^+^ T cells on the benign prognosis of patients with CRC, demonstrating their anti-tumor capacity (40). Studies on neutrophil heterogeneity in mice are applicable to humans, highlighting their importance in cancer research.

Chemokines have emerged as key regulators in tumor immunity, with roles in both promoting and suppressing cancer becoming clearer (41). CXCL1, a significant chemokine, plays a crucial role in neutrophil aggregation. Our analysis of CXCL1 in the Kaplan-Meier Plotter database revealed that its high expression in CRC patients corresponds to higher survival rates. Numerous studies have explored the interaction between CXCL1 and neutrophils in cancer contexts. For example, Ogawa (42) demonstrated enhanced CXCL1 production and subsequent neutrophil recruitment in CRC cells following SMAD4 gene knockdown. Additionally, Tosti (43) showed that IL22-induced secretion of CXCL1 promotes anti-tumor neutrophil chemotaxis to the TME in CRC cells. Our experiments align with these findings, indicating that YHJD treatment significantly increases CXCL1 levels in both liver tissue and plasma. As CXCL1 exerts a potent chemotactic effect on neutrophils, the observed increase in CXCL1 levels likely contributes to enhanced neutrophil recruitment to the liver. These neutrophils, particularly the N1 subtype, possess pro-inflammatory and immune-stimulating properties, collectively inhibiting liver metastasis of CRC.

Chinese medicine is increasingly used as an adjunct to tumor treatment, particularly for prevention, metastasis, and recurrence, owing to its multitargeting and multifunctional properties (44). The adjunctive role of Chinese medicine in tumor prevention and treatment has been well established. Chinese medicine has demonstrated efficacy in treating liver metastasis of CRC by affecting immune cells through multitargeting, regulating tumors, and enhancing patients’ immune function (45–47). Given the promising inhibitory effect of YHJD on CRC liver metastasis in clinical practice, its holistic approach aligns well with the principles of Chinese medicine. By leveraging YHJD’s multitargeting approach, we can potentially enhance the body’s natural defenses against cancer progression, particularly in the context of CRC liver metastasis. This holistic approach addresses not only the tumor itself but also considers the overall well-being of the patient, making it a valuable addition to conventional cancer treatment strategies. Moreover, the analysis of differential genes in proteomics identified Chil3, which has been implicated in the chemotactic activity of granulocytes. It has been demonstrated that both Chil3 and CXCL1 act on the chemotactic activity of granulocytes (48). Therefore, Chil3 probably assists CXCL1 in its function of chemotaxis to neutrophils. Overall, these findings underscore the complex interplay between immune modulation, metabolic regulation, and TME remodeling in the context of YHJD treatment for CRC liver metastasis.

Fas, also known as Apo1 or CD95, belonging to the tumor necrosis factor (TNF) receptor family, plays a significant inhibitory role in tumor formation and progression (49). Analysis of the Kaplan-Meier Plotter database revealed that high Fas expression in CRC patients corresponded to a higher survival rate. The Timer database showed a positive correlation between Fas and neutrophil immune infiltration. Therefore, we analyzed changes in Fas in liver tissue and conducted GO and KEGG analyses. Our analysis of liver tissue demonstrated that YHJD treatment elevated Fas levels, indicating a potential mechanism through which YHJD modulates the immune response to CRC. Fas serves as a marker gene for N1 neutrophils, reflecting changes in N1 neutrophils that contribute to a favorable immune environment, which is conducive to suppressing tumor progression (50). To further confirm the feasibility of YHJD for treating CRC liver metastasis through neutrophil chemotaxis, we performed KEGG enrichment analyses of differential genes in proteomics and transcriptomics. The results indicated pathways such as valine, leucine, and isoleucine degradation, sulfur metabolism, propanoate metabolism, butanoate metabolism, D-amino acid metabolism, and others. Among these, leucine and isoleucine significantly impact tumorigenesis in various human malignancies (51, 52). Studies have shown that altering amino acid metabolism can affect tumor growth and progression (53, 54). Beall (55) found that inducing amino acid residues can affect the expression of valine and leucine, thereby enhancing the chemotactic activity of neutrophils. Yochiro (56) analyzed the components of neutrophil chemokines and found that isoleucine and leucine are related to the formation of neutrophil chemokines. Notably, alterations in amino acid metabolism can affect tumor growth and progression, with increased levels of amino acids enhancing the anti-cancer immune response (57). Conversely, defects in amino acid metabolism promote tumor metastasis to different organs due to the destructive effects of high amino acid concentrations on tumor cell migration and invasion (58). Additionally, sulfur metabolism has been strongly associated with CRC development, further highlighting the potential of YHJD in inhibiting liver metastasis by regulating these metabolic pathways (59). Therefore, YHJD could inhibit liver metastasis of CRC by regulating the degradation of valine, leucine, and isoleucine, as well as sulfur metabolism.

Our experiment aims to uncover the therapeutic mechanism of YHJD in CRC liver metastasis patients and interpret it from the perspective of neutrophils to verify the adjunctive role of traditional Chinese medicine in liver metastasis treatment. However, the research on the effectiveness of YHJD on neutrophils will not be limited to this. Although the doses we used are supported by clinical data and previous studies, it is still necessary to observe the effects of YHJD on liver metastases in mice at different doses, as well as the chemotactic effects on neutrophils, especially N1 neutrophils. Besides, more comprehensive experiments are needed to validate the time-effect relationship of YHJD on neutrophils. We will conduct further experiments to determine the effects of YHJD on neutrophil plasticity and maintenance of N1 neutrophil function at different times of YHJD treatment. Metabolic shifts in the microenvironment affect immune cells to produce different functions, and neutrophils have been shown to respond positively to changes in the local environment (60). After YHJD promotes the chemotaxis of N1 neutrophils into the liver, the effects of YHJD on the liver microenvironment and whether N1 neutrophils regulate metabolic pathways for tumor suppression remains to be thoroughly explored. Therefore, the study of YHJD’s mechanism for treating liver metastasis from CRC is ongoing and requires further investigation.

## 5 Conclusion

In summary, our research suggests that YHJD can enhance the chemotaxis of N1 neutrophils to liver tissue by influencing the chemokine CXCL1, thereby potentially reducing CRC metastases. Bioinformatics analysis further illustrated the high correlation of CXCL1 and Fas with neutrophil immune infiltration in the TME. This study may provide new anti-tumor drugs for the treatment of CRC liver metastases. The potential metabolic pathways of N1 neutrophils after entering the liver also offer a good idea for the subsequent study of YHJD for the treatment of CRC liver metastases. However, the key active components and detailed mechanisms of the inhibitory effect of YHJD on CRC liver metastasis need to be further revealed in subsequent studies. In addition, YHJD affects some metabolic pathways to some extent, including sulfur metabolism, propanoate metabolism, which also needs further investigations.

## Data Availability

The data presented in the study are deposited in the BioProject in National Genomics Data Center, China National Center for Bioinformation / Beijing Institute of Genomics at https://ngdc.cncb.ac.cn/, accession number: PRJCA036419.
